# Multiple Imputation Approaches Applied to the Missing Value Problem in Bottom-Up Proteomics

**DOI:** 10.3390/ijms22179650

**Published:** 2021-09-06

**Authors:** Miranda L. Gardner, Michael A. Freitas

**Affiliations:** 1 Ohio State Biochemistry Program, Chemistry and Biochemistry, The Ohio State University, Columbus, OH 43210, USA; gardner.207@osu.edu; 2 Cancer Biology and Genetics, Wexner Medical Center, The Ohio State University, Columbus, OH 43210, USA

**Keywords:** bottom-up proteomics, label-free quantitation, spectral counting, peak intensity, missing value imputation

## Abstract

Analysis of differential abundance in proteomics data sets requires careful application of missing value imputation. Missing abundance values widely vary when performing comparisons across different sample treatments. For example, one would expect a consistent rate of “missing at random” (MAR) across batches of samples and varying rates of “missing not at random” (MNAR) depending on the inherent difference in sample treatments within the study. The missing value imputation strategy must thus be selected that best accounts for both MAR and MNAR simultaneously. Several important issues must be considered when deciding the appropriate missing value imputation strategy: (1) when it is appropriate to impute data; (2) how to choose a method that reflects the combinatorial manner of MAR and MNAR that occurs in an experiment. This paper provides an evaluation of missing value imputation strategies used in proteomics and presents a case for the use of hybrid left-censored missing value imputation approaches that can handle the MNAR problem common to proteomics data.

## 1. Introduction

Improvements in mass spectrometry instrumentation and methodologies, along with a growing interest in the exploration of multi-omics, incorporating proteomics with genomics for targeted therapeutics, have led to a generation of large, high-density datasets and introduced a new confounding factor in data analysis of data-dependent acquisition (DDA) methods: missing values [1,2,3,4]. Missing values in mass spectrometry-based proteomics data analysis can range from 5 to 50% in any given replicate for observed peptide abundances. Two approaches to deal with missing values are (1) removing peptides/proteins that have insufficient samples for analysis or (2) imputing values as placeholders for the missing values [5,6,7,8,9,10,11,12]. The former approach may be acceptable when analyzing a small number of samples with similar proteomic profiles and few missing values. However, the latter imputation approach, when appropriately applied, can avoid unnecessarily excluding data from analysis.

The most common sources of missing values in proteomics experiments are (1) the biology and/or technical sample preparation, (2) actual presence below the instrument’s limit of detection (LOD) threshold and (3) presence above the LOD but an error in data preprocessing [13]. Furthermore, missing values in proteomics data can be classified into one of three categories: missing completely at random (MCAR), missing at random (MAR) or missing not at random (MNAR) [14]. MCAR is independent of the data and observed values, much as its name suggests, and is likely to occur across the entire distribution of data. This type of missingness originates from inaccurate instrumentation or oversight in experimental sample preparation. MAR covers a wider range than MCAR, and the missingness is due to conditional dependency on observed values. This class of missing values can arise when the peptide sequence is mapped incorrectly or software erroneously assigns shared peptides to precursors leading to misidentification in some samples and missing values in others.

MAR methods considered for this study include *k* nearest neighbors (*k*NN), singular value decomposition (SVD) and maximum likelihood estimate (MLE). The *k*NN algorithm imputes missing values to the protein of interest from proteins with similar expression profiles. The missing value is estimated from a weighted average of the *k* closest proteins to the protein of interest [15]. The SVD method converts the dataset to eigenproteins, a principle component protein expression matrix. This algorithm fills in missing values with row averages and performs consecutive iterations with eigenprotein regression until the total change in the matrix falls below 0.01 [15]. MLE assumes that data are a function of an unknown parameter θ. The imputed value is a random draw of the MLE of θ that maximizes the probability of the observed data [6,16,17].

MNAR is a non-ignorable case of missing values contrasting the previously mentioned MAR, where the missing values arise as a direct relationship with the data. MNAR may result from experimental effects in proteomics such as (1) enzyme miscleavages, (2) true presence/absence (as seen in immunoprecipitation (IP) when comparing a treatment to an immunoglobulin control (IgG)) and (3) instrumentation effects (dynamic range or LOD occurring when peptide measurements are low in abundance compared to background noise or constitute low ionization efficiency). Because the missingness is influenced by the low abundant nature of these values, this category of missing values is considered left-censored where the distribution of values (if present in the data) would fall on the left tail of the total observations in the dataset.

Non-ignorable MNAR methods considered for this study include deterministic minimum (MinDet), probabilistic minimum (MinProb) and quantile regression imputation of left-censored data (QRILC). MinDet replaces each missing value with the smallest detectable intensity across the entire dataset or observed within each sample [18,19]. Similar to MinDet, MinProb also replaces missing values with the smallest value, but only after the data are first centered on the MinDet value. Then, the replacement for the missing value is a random draw from the Gaussian distribution [6,20]. The QRILC approach utilizes quantile regression to construct a truncated distribution from the leftmost tail of the data, and the missing values are then replaced with random draws from this reduced allotment [9].

The current literature on missing values imputation has explored the type of imputation (single/multiple), nature of missingness (MAR, MCAR or MNAR) applied across entire datasets, statistical algorithms to determine differentially expressed/enriched proteins (DEP) (reviewed further in [7,8,11,21,22,23,24]) and development of software tools to visualize the results (discussed further in [25,26,27,28,29,30,31]). Single imputation strategies work well with datasets that are very similar in nature (low number of missing values), simulated missing values and very large time course studies containing multiple time points and biological replications [6,24,32,33]. However, as the number of missing values increases, it has been suggested that a single value estimate is not capable of generating the missing value accurately or capturing the variability. Additionally, a value too small or too large from the “true value” will heavily influence the downstream statistical analysis [10,34].

The multiple imputation (MI) approach addresses the single value estimate concern by performing consecutive iterations of a chosen method to generate *m* imputed datasets, followed by a selection step that combines every *m* imputations into a final dataset for downstream analysis. These methods work well with small datasets, but the imputation method and modeling approach are data-dependent, sensitive to parameter selection and may need further optimization for effective implementation [10,35,36]. In contrast, multiple imputations in multi-factor analysis (MI-MFA) were successfully applied to large datasets without a priori knowledge of the missingness. However, the MI-MFA approach assumes that missing values are MAR, require good donor-recipient matches and may introduce bias if the sample size is too small, yielding too few donors in the donor pool [34].

As it is often difficult to determine the main contributor to missing values, approaches that combine MAR and MNAR methods are important to consider. Here, we provide a comparison of several different combinations of workflow imputation methods available via the imputeLCMD package in R [6,37] and offer insight into the most appropriate workflow and method for handling the missing data problem in proteomics. We further demonstrated that across increasing numbers and type of missingness, the MNAR/MAR MI SFI-hybrid approach consistently outperformed other methods, as evidenced by the percentage of significant proteins captured in the simulated/amputed data (with one exception), along with the accurate calculation of logFC protein expression values and *q*-value significance in DEP comparisons when applied to actual proteomics data.

## 2. Results

### 2.1. Imputation with Simulated Dataset

As described in the methods section, data were simulated from the MDA-MB-468 dataset by treatment group using the mean and standard deviation of the observed values. This resulted in a dataset consisting of 3807 proteins with three or six total observations (three missing values in one treatment group or zero missing values). The dataset was used to establish a baseline for comparing imputation methods wherein we know the source of missingness. The total missingness was 9.2%, including 12.2% in the glucose deprivation (GD) treatment group and 6.2% in the high glucose (HG) treatment group (Table 1 and Table S1). After 25 consecutive iterations and using a threshold of 0.05 for significance cut-off, the following DEP lists were observed: 2570 significant proteins when using *k*NN, 1794 for MLE, 2494 for SVD, 2647 for MinDet, 2527 for MinProb, 2509 for QRILC and 2537 for SFI-hybrid (Table 1 and Table S1). The simulated dataset and resulting DEP analyses signify the ground truth for this data.

### 2.2. Type of Amputation and Increasing Missingness Influences on Significance

**Figure 1:**
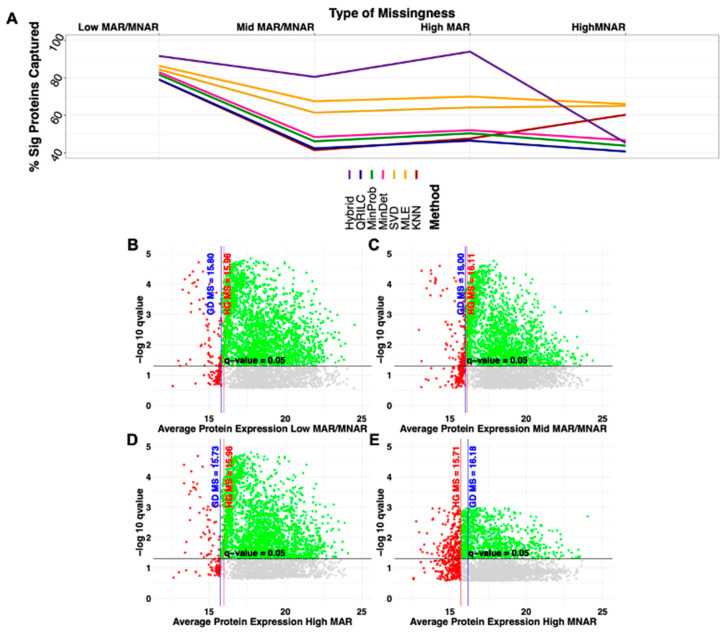
Summary of imputation method performance with increasing missingness (amputation type) in simulated data and protein expression profiles in hybrid. (**A**) The percentage of significant proteins remaining from the original simulated data as missingness increased for each of the imputation methods. (**B**–**E**) Significance versus protein expression for each of the amputed datasets following the hybrid imputation method. Proteins in green represent significant proteins with expression or imputed expression above the lowest (leftmost) MNAR model selector value, while non-significant above the MNAR model selector value are in gray. Those in red are imputed below the lowest MNAR model selector value and are significant above the black line (*q*-value < 0.05) and not significant below the black line.

To investigate how missingness alters the performance of MAR, MNAR and hybrid imputation methods and downstream statistics when attempting to determine significance, datasets containing different missing value combinations were generated from the MDA simulated data using the ampute function from the mice package in R. Missing value combinations are data patterns of variables containing missing values and variables that remain complete as determined primarily by two user-provided factors: the missingness mechanism (MAR or MNAR) and the proportion of missingness. In this study, four different types of amputation were performed on the simulated data to introduce more missing values: low missing values (Low MV, 0.2: 0.2 MAR: MNAR), mid missing values (Mid MV, 0.5: 0.5 MAR: MNAR), high MAR (0.8: 0.2 MAR: MNAR) and high MNAR (0.2: 0.8 MAR: MNAR). Following amputation, total missingness increased by 3.3% in Low MV, 8.8% in Mid MV, 8.3% in high MAR and 8.5% in high MNAR (distribution can be found in Table S1). The percentage of significant proteins captured following imputation of the amputed datasets ranged from 40.1% in the High MNAR with the QRILC method to 94.1% in the High MAR with the hybrid method (Table 1, Figure 1a and Table S1).

With the exception of High MNAR, the hybrid method outperforms all other imputation methods in terms of capturing the most (highest percentage of) significant proteins from the original simulated data (Figure 1). The High MNAR amputation data resulted in the highest proportion of missing values imputed below the MNAR model selector threshold when compared to the other three amputation datasets (Figure 1b–e).

### 2.3. Imputation with Small Dataset of Similar Proteomic Profiles

**Figure 2:**
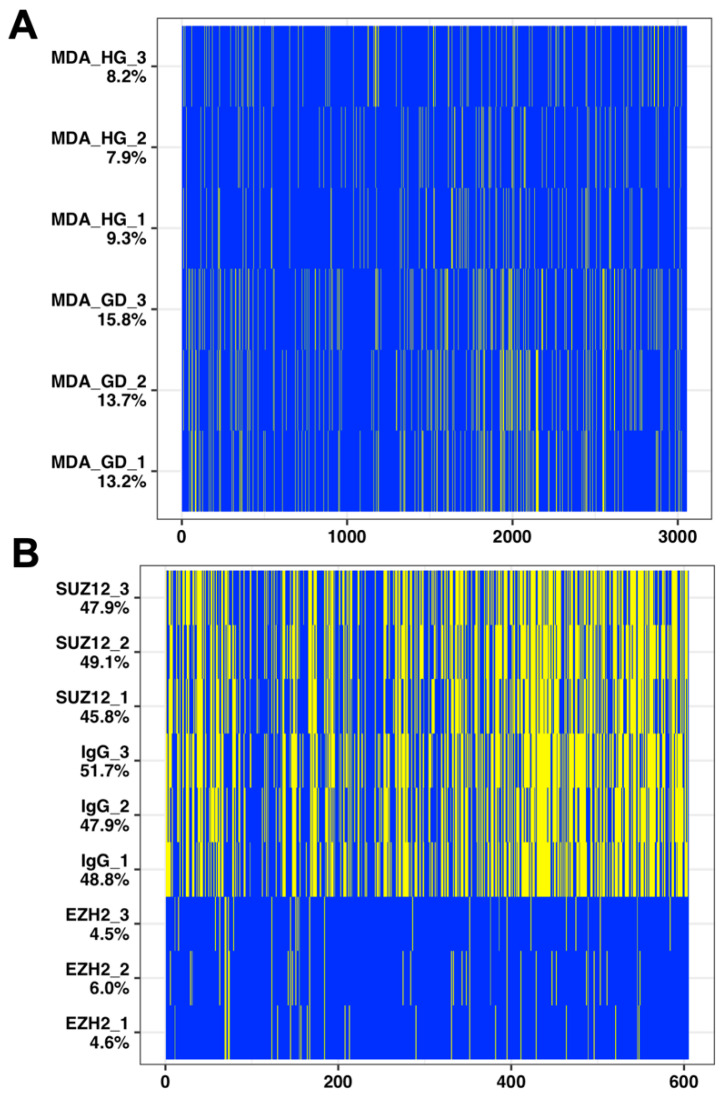
Distribution of number of missing values in the two datasets analyzed for this manuscript. The MDA data (**A**) are very similar in terms of the number of missing values (depicted as yellow lines) ranging from 8 to 14% relative to observed values (depicted as blue lines), while the IP data (**B**) demonstrates a presence/absence dataset with missing values ranging from 5 to 52%.

The PXD013966 dataset consisted of triple-negative breast cancer cells MDA-MB-468 exposed to high glucose (*n* = 3) or glucose deprivation (*n* = 3) for 48 h prior to sample preparation and analysis for bottom-up mass spectrometry. This whole cell proteomics experiment is representative of a small dataset with treatment and control in biological triplicate, where most of the proteins are present, and changes in protein expression levels are detected. The number of missing peptide values ranged from 8 to 16% across samples with a similar protein expression as seen by the distribution pattern of observed and missing peptides (Figure 2a).

**Figure 3:**
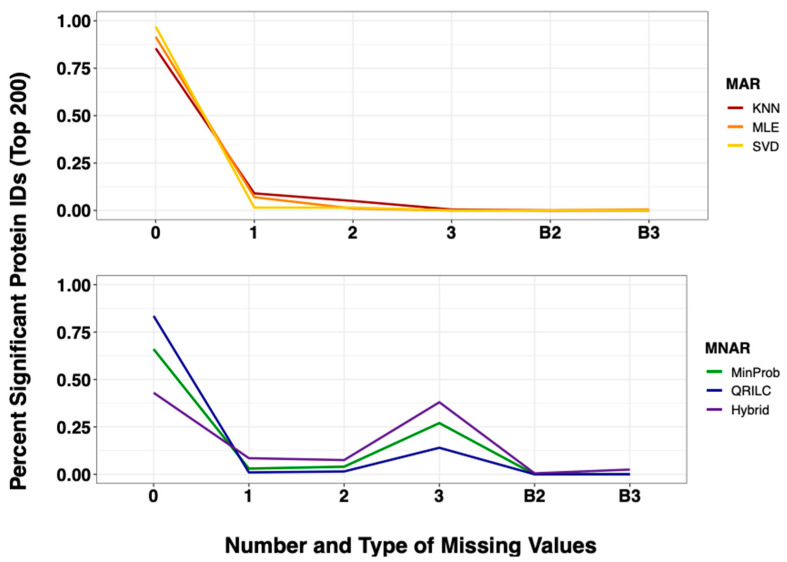
Frequency of missingness across MAR (**Top** Panel) and MNAR (**Bottom** Panel) imputation methods with the top 200 significant proteins in MDA data. After 25 consecutive iterations, each imputation dataset was rank-ordered by *q*-value mean. The top 200 proteins from each method were binned according to the type of missingness: 0, 1, 2 or 3 missing values in one sample group or a combination of 2 or 3 in both groups (B2, B3).

Following filtering of contaminants (proteins present in cRAP and MaxQuant Databases), the data were further filtered to reduce sparse data from the analysis. Sparse data are proteins with an abundance <2^15^ in 50% of the samples. After filtering, 3165 total proteins remained in the dataset. After performing 25 consecutive imputations, calculated logFC values remained fairly stable, and the ranges of protein expression values were not influenced by the number/type of missing values in the methods examined (Figure S1). By using a threshold of 0.05 for significance cut-off, the following DEP lists were observed: 1186 significant proteins when using *k*NN, 1180 for MLE, 1106 for SVD, 1299 for MinDet, 1312 for MinProb, 1292 for QRILC and 1669 for SFI-hybrid. Each imputation method DEP list was ranked by *q*-value mean, and the top 200 DEPs from each method were classified into the number and type of missingness as follows: missing 0, 1, 2, or 3 values in one sample group (0, 1, 2, 3) or missing any combination of 2 or 3 values in both groups (B2, B3). With the exception of the SFI-hybrid method, the majority of the proteins determined to be significant were those with all observations present (no missing values; far left, 0 missing values; Figure 3 and Figure S2).

The SFI-hybrid approach was the only method where missing values did not have a detrimental effect on downstream statistics as approximately 80% of proteins deemed significant were split equally between the 0 and 3 missing value groups (Figure 3 and Figure S2). Furthermore, the SFI-hybrid approach demonstrated the most reproducibility in terms of the standard deviation of logFC expression and maintaining *q*-value significance in all protein identifications (Figures S1 and S2).

**Figure 4:**
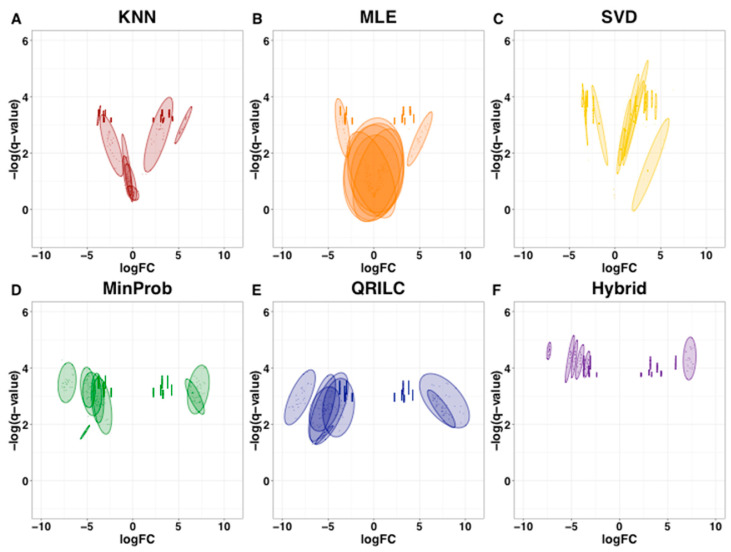
(**A**–**F**) Spread plots of −log *q*-value vs. logFC for merged top proteins across all imputation methods for MDA-MD-468 data. Following 25 consecutive iterations, each dataset from each imputation method was ranked by *q*-value mean. The top 10 proteins from each method were pooled into a top protein list (Supplemental Table S2) and plotted here. The ellipse represents a 95% confidence interval. Note MinDet is not shown as this method imputes the same value after each iteration.

To further examine the reproducibility and accuracy of the methods in terms of handling missing values and imputation, a common top protein list was constructed. The top 10 proteins from each imputation method were merged to a single list, retaining only the unique protein IDs. This list contained 19 total proteins with the following distribution of missing values: 0 missing values—11 proteins, 1 missing value—1 protein, 2 missing values—1 protein and 3 missing values—6 proteins. This list of 19 proteins was applied as a filter to each of the seven individual DEP lists in order to extract the mean logFC, mean −log10 *q*-value, and standard deviations of both measurements. These data were visualized as spread plots in Figure 4. The SFI-hybrid performed best, followed by the MNAR methods (Figure 4 and Table S3). The MAR MLE method performed the worst, with kNN and SVD falling in the middle (Figure 4 and Table S3).

### 2.4. Imputation Influences with Increasing Number and Type of Missing Values

The PXD004462 sample set consists of NTERA2 (NT2) pluripotent embryonic carcinoma cells treated with retinoic acid for 8 days to induce neuronal differentiation. Therefore, we expected the nuclei that were isolated for immunoprecipitation tandem mass spectrometry (IP-MS/MS) with EZH2 (*n* = 3), SUZ12 (*n* = 3) and IgG (*n* = 3) to have a true presence/absence in protein profiles. The number of missing values in this dataset ranged from 8 to 52% (Figure 2b). Data were ranked by *q*-value mean following multiple imputations and classified as described in the methods below.

Following filtering of contaminants (proteins present in cRAP and MaxQuant Databases), the data were further filtered to reduce sparse data from the analysis. Sparse data are proteins with an abundance <2^15^ in 50% of the samples. After filtering, 601 total proteins were retained in the EZH2 IP dataset. With a *q*-value threshold of 0.05, DEP lists included: 338 significant proteins when using *k*NN imputation, 348 for MLE, 375 for SVD, 326 for MinDet, 179 for MinProb, 198 for QRILC and 365 for SFI-hybrid. With the same filtering criteria as mentioned above, the SUZ12 IP contained 350 proteins. The resulting DEP lists included: 119 significant proteins when using *k*NN, 97 for MLE, 102 for SVD, 127 for MinDet, 120 for MinProb, 111 for QRILC and 187 for SFI-hybrid.

**Figure 5:**
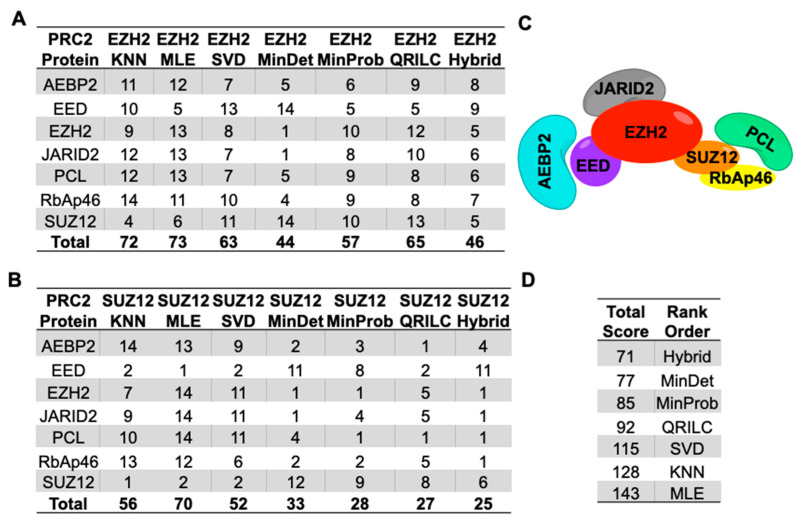
Rank ordering of PRC2 component IPs. Each component of the PRC2 complex identified in EZH2 (**A**) and SUZ12 (**B**) IPs were rated (1–14) according to position in the rank-ordered protein lists (Supplemental Table S1). (**C**) Canonical human PRC2 protein complex, adapted from Margueron, R., 2011, *The Polycomb Complex PRC2 and its Mark in Life*. Nature, 2011. 469(7330): p. 343–9 (**D**) Total ratings and overall ranking of each method.

Approximately one-half of the proteins determined to be significant contained all observations (no missing values) in every imputation method for both IP experiments (Figures S5 and S10). The increasing number of missing values resulted in a wider range of logFC protein expression values across imputation methods for the EZH2 and SUZ12 IPs (Figures S3 and S8) than seen in the MDA data. A common top protein list was generated as described above. This merged top protein list for EZH2 IP consisted of 21 proteins (3 missing values—3 proteins, 2 missing values—3 proteins, 1 missing value—3 proteins, and 0 missing value—12 proteins) while the SUZ12 IP list contained 23 total proteins (3 missing value—11 proteins, 1 missing value—4 proteins, and 0 missing value—8 proteins). The SFI-hybrid method performed better with an increasing number of missing values, as evidenced by the narrow ellipses in the spread plots showing the tighter ranges of values for logFC and −log10 *q*-values (Figures S3, S4 and S6–S9). These data illustrate the impact the missingness and appropriate choice of imputation method can have on the range of values obtained in DEP analysis. The combined protein ranks were determined as described in the methods section and used to evaluate the relative rank of each of the canonical PRC2 complex members from both the EZH2 and SUZ12 IPs. These rankings show that the SFI-hybrid method outperformed all other imputation methods, followed by the MNAR methods (MinDet, MinProb, QRILC), SVD, *k*NN and MLE (Figure 5a,b,d and Table S1).

## 3. Discussion

Mass spectrometry experiments are prone to MNAR and MAR due to the nature of the prevailing bottom-up proteomic experimental design. Data-dependent and data-independent experiments prioritize observations of higher abundance species that inherently induce stochasticity at the low end of detection. Targeted mass spectrometry experiments prioritize observations based on a few selected targets, providing less overall coverage but a better sampling of peptide abundances. DDA experiments can employ dynamic exclusion in an attempt to improve the sampling of low abundance species. However, these scheduled prioritizations can also induce missing values when peaks are sampled early or late, leading to poor quality MS2 scans that may not be matched to a peptide sequence. This paper focuses solely on the evaluation of how different missing value imputation methods function in label-free quantitative data analysis across a range of missingness specific to DDA bottom-up proteomics.

We chose to work with the methods within the imputeLCMD package because they span a wide range of imputation methods in an accessible R package (described in more detail in [6,37]). Additionally, the imputation wrappers and functions can be altered to allow a multiple imputation strategy, and the model selector can be changed for all MAR or all MNAR imputation patterns and, importantly, contains the function impute.MAR.MNAR to simultaneously model the data in a combinatorial manner. As described here, we first investigated how missingness can influence the statistics in DEP analyses by evaluating the performance of seven separate imputation methods with simulated and amputed data. We then applied the same imputation methods to model two dissimilar sets of data to be all or mostly all MAR, MNAR or a combination of the two.

The fixed random seed generator included in the imputation wrappers and functions was changed to allow a multiple imputation approach to address issues discussed in the introduction. Further, for *k*NN and SVD, this meant that the algorithms would be limited to the iteration number of the loop (ranging from 1 to 25) for the nearest neighbor or principal component calculations of the imputation variable that would replace the missing value. For example, iteration 10 of the multiple imputation loop would allow either 10 nearest neighbors or principal components to draw potential imputation variables from. In the case of all MARs, the model.selector argument in the impute.MAR function was converted such that all rows in the dataset would be flagged strictly MAR. Our aim was to develop a robust technique that would be applicable across many types of data with transparency, accuracy and reproducibility.

Here, we propose utilizing a combinatorial MAR/MNAR, or SFI-hybrid, with a typical model selector but performing imputation separately for each treatment or group of data. This approach is shown to most accurately model data regardless of type. This observation is not unexpected in that even for highly similar datasets with replication and few missing values, the left-censored nature of proteomics data is well suited to hybrid imputation methods.

The SFI-hybrid method considerably outperforms the other imputation methods with most instances of amputed data by consistently capturing 80.5% of the simulated significant proteins with the Mid MV instance, 91.7% in the Low MV instance and 94.1% in the High MAR instance (Figure 1a and Table S1). However, this method was only able to capture 45.3% of the simulated significant proteins in the High MNAR instance. Specifically, in this instance, the MAR methods (*k*NN, MLE, SVD) equally outperformed the MNAR methods (MinDet, MinProb, QRILC) and the SFI-hybrid by capturing 60.3%, 66.1% and 65.1% of the simulated significant proteins, respectively (Figure 1a and Table S1). Further examination of the amputed data demonstrated the High MNAR instance had the largest number of missing values imputed below the lowest MNAR model selector value, resulting in a drastic change in significance as evidenced by the smaller *q*-value range (Figure 1b–e). These observations in the High MNAR instance suggest the left-censored techniques may not be performing as well because the original data consist of more MAR values or the data, following amputation, contain too few observations to model the data properly.

Analysis of the MDA proteomics dataset revealed the SFI-hybrid method maintained the same level of significance across all missing value types with the smallest standard deviations (Figures S1 and S2). Additionally, there was a negative trend toward non-significance with the increasing number of missing values in all methods, excluding the SFI-hybrid (Figure S2). To examine the top significant proteins in the MDA dataset, we binned each protein into the number (0, 1, 2, 3) and type of missing value (B for missing in both treatments). As expected, fewer proteins were designated as significant when there were missing values present in both treatment groups (Figure 3 and Figure S2). We found all methods, other than the SFI-hybrid, favored complete cases with greater than 60% of significant identifications containing no missing values. This observation suggests that either the methods have a bias to choose complete cases, or the algorithms are imputing values too close to the observed to be considered significant.

To further investigate the performance of the missing value imputation methods for preserving accurate logFC and *q*-values, spread plots were constructed as described in the results and methods sections. The SFI-hybrid and MNAR methods performed better at preserving the significance level than the MAR (Figure 4 and Table S2) as all proteins were found to be significant, and the confidence ellipses representing the standard deviation were located above the threshold cut-off (-log10 (*q*-value) > 1.3). This is expected as MNAR methods are designed specifically for the low-abundant nature of these absences and impute the left-censored data appropriately. The MLE method appears to have performed the worst, as six of the 19 proteins were not significant (Table S2). Upon closer examination of the data, this only occurs when there are three missing values and can be explained by the large standard deviation after multiple imputations. The four proteins not significant with the *k*NN method and with opposite logFC values in the SVD method were also from the three missing value types (Table S2). When investigating proteins with three missing values that are imputed using *k*NN or SVD, we observe that logFC is highly variable (characterized by large standard deviations) and can change direction as well (oscillate between positive and negative values). We would caution the choice to use a single imputation strategy with these two methods and, instead, encourage the use of 15–16 nearest neighbors with *k*NN and 6–7 principal components with SVD to ensure the algorithms are performing optimally for missing value imputation (having enough donor information to impute values that are not negatively influencing the downstream statistics). Altogether, these results imply the missing data are not all MAR or all MNAR and imputation should be performed with a strategy to reflect that.

A similar analysis was performed with the EZH2 and SUZ12 IPs (as mentioned above with the MDA-MB-468 data). The SFI-hybrid method maintained a similar level of significance across all missing value types with the smallest standard deviations in both IPs (Figures S4 and S9). The range of logFC values varied in the SUZ12 IP (Figure S8) and is attributed to a large amount of missingness when compared to EZH2 IP. The more stringent SUZ12 IP resulted in fewer observed values leading to under-represented variation after imputation. Interestingly, there was an opposing trend with increasing missingness in the EZH2 IP; logFC values shrank towards zero when using MAR methods and expanded when imputing with MNAR methods (Figure S3). All imputation methods, excluding the SFI-hybrid, trended toward non-significance and large variance across *q*-value as missingness increased (Figures S4 and S9). As mentioned above, these observations offer further support that the algorithms for MAR methods are imputing values too close to the observed to be considered significant or have an inherent bias to favor complete cases.

Once the top 100 significant proteins in the IPs were binned according to missingness, we found the SFI-hybrid significant protein list evenly distributed across missingness with approximately 40% in complete cases or three missing values (Figures S5 and S10). In the EZH2 IP, all methods excluding the SFI-hybrid favored complete cases. Excluding MLE, when three missing values were present, all other methods demonstrated a statistical difference in the pair-wise comparison of EZH2 to IgG (Figure S5). In the case of SUZ12 to IgG, the *k*NN and MLE MAR methods favored complete cases while SVD and MNAR modeled the same trend as seen with the SFI-hybrid in the EZH2 IP (Figure S10).

Missing values and type of imputation, specifically when using MAR methods *k*NN and MLE, did influence the downstream statistics when considering the top protein lists generated for both sets of IPs (described in the methods section; Tables S3 and S4). Further, the SFI- hybrid method is most consistent with imputing values for both sets of IPs, as seen by the tight confidence ellipses (Figures S6 and S7). The *k*NN and MLE methods were the worst performers with the SUZ12 IP data; the top protein list was characterized by the largest variances (Figure S7) and the greatest number of non-significant protein identifications at 7 and 11 when imputing with these approaches (Table S4). Additionally, two of the seven and five of the seven PRC2 complex proteins were determined as not significantly enriched in the *k*NN and MLE methods. It is interesting to note that these were instances where three missing values occurred. These observations suggest the missing data are not all MAR or all MNAR, and caution should be taken to choose an imputation strategy that appropriately models the data, such as the MI SFI-hybrid approach.

To determine the overall performance of the methods presented in this study, we focused on the IP dataset since it is more representative of a bottom-up presence/absence proteomics experiment with values MNAR. We decided to examine the data from both IPs in a combinatorial manner because we did not want the results biased from the large range of missing values or the non-specificity of the EZH2 antibody (Figure 2b). The canonical PRC2 complex (Figure 5c) consists of seven proteins: AEBP2, EED, EZH2, JARID2 (JARD2), RbAp46 (RBBP7), SUZ12 and PCL family. We arbitrarily chose the PCL protein MTF2 for our ranking analysis. Following the MI strategy and ranking by *q*-value mean, we recorded the overall position (in ascending order, lowest *q*-value to highest) for each of the PRC2 components for each IP across each method separately (Table S1). This information was then used to determine the performance of each imputation strategy as described in the methods and results sections. This ranking approach demonstrated MinDet performed best, followed closely by SFI-hybrid for the EZH2 IP data. The MNAR and SFI-hybrid methods outperformed the MAR methods when dealing with large amounts of missing data as in the SUZ12 IP as noted by the rankings of proteins in the canonical PRC2 complex with the exception of EED (Figure 5a,b). Upon closer examination of the dataset, we observed EED was the only protein in the complex observed in all three conditions (0 missing values). This observation suggests the algorithms for MAR methods have an inherent bias to emphasize significance with instances where there are complete cases (all observations). Overall, we determined the SFI-hybrid had the best ranking from the summed ratings of the canonical PRC2 components, followed by MNAR and MAR methods (Figure 5d).

## 4. Materials and Methods

### 4.1. Proteomic Datasets

#### 4.1.1. Glucose Deprivation

The RAW Thermo files for the analysis of this manuscript were downloaded from the PRoteomics IDEntifications database (PRIDE): PXD013966. This dataset originated from Lee et al. [38], in which triple-negative breast cancer cell line MDA-MB-468 was exposed to high glucose (25 mM, *n* = 3) or glucose deprivation (0 mM, *n* = 3) for 48 h. Cells were lysed as described in the methods section [38]. Protein from each sample was prepared for bottom-up proteomics by reduction/alkylation with DTT and iodoacetamide. Mass spectra were collected on a Q Exactive™ (Thermo Fisher Scientific, Waltham, MA, USA) operated in data-dependent acquisition (DDA) mode. In the Lee et al. publication, the authors identified 2525 proteins and determined 681 DEPs (*p*-value < 0.01). For this investigation, RAW mass spectrometry files were converted, searched and quantified as described in the methods sections below. Samples were grouped according to treatment (high glucose or glucose deprivation), and differential expression analysis was carried out to determine if any proteins were significant (*q*-value < 0.05). This dataset was used as the baseline data for the creation of a ground truth simulated data set (assuming all significant differences are true positives). It was also used to reevaluate and confirm findings and trends seen in the simulated/amputed data described below and further investigate the way different imputation methods alter the downstream statistical calculations and false discovery rate (FDR).

#### 4.1.2. Pluripotent Cell Differentiation

The RAW Thermo files for the analysis of this manuscript were downloaded from the PRIDE database: PXD004462. The NTERA2 (NT2) dataset consisted of proteins quantified and identified after IP of polycomb repressive complex 2 (PRC2) subunits EZH2 (*n* = 3) and SUZ12 (*n* = 3) along with IgG control (*n* = 3) [39]. Detailed sample preparation is described in the methods section of Oliviero et al. [39]. Briefly, NT2 cells were left undifferentiated or treated for 8 days with retinoic acid (10 μM) to induce differentiation and nuclei from both cell lines were isolated for IP with EZH2, SUZ12 or IgG. Proteins from each pull-down were eluted from beads and prepared for bottom-up proteomics analysis by tryptic in-solution digestion. Mass spectra were collected on a Q Exactive™ (Thermo Fisher Scientific) operated in DDA mode. In the Oliviero et al. publication, 366 candidate EZH2-interactors and 191 candidate SUZ12-interactors were identified (false-discovery rate (FDR) threshold = 0.05). For this investigation, RAW mass spectrometry files were converted, searched and quantified as described in the methods sections below. Samples were grouped according to experimental immunoprecipitation conditions (EZH2, SUZ12 or IgG) and two pair-wise differential expression analyses were carried out to determine if any proteins were significant (EZH2 vs. IgG and SUZ12 vs. IgG, *q*-value < 0.05). This dataset was used to evaluate the effect of imputation methods on data with a high occurrence of MNAR.

### 4.2. Database Searching and Label-Free Quantitation (LFQ)

Mass spectra from both sample sets were searched with the OpenMS platform (v 2.5.0) and X!Tandem search engine against a reviewed UniProt human proteome (19 May 2019) containing the cRAP and MaxQuant contaminant FASTAs with the following parameters: full trypsin digest, 2 missed cleavages, variable modifications (oxidation of methionine +15.99491, carbamidomethyl of cysteine +57.02146), precursor (MS1) mass tolerance 20 ppm and fragment (MS2) mass tolerance 0.02 Da. PSM rescoring was completed with Percolator, and protein inference was performed with FIDO across all samples, setting peptide and protein FDR to 0.05.

### 4.3. Generation of Simulated and Amputed Datasets

The MDA-MB-468 data were used as the basis for creating simulated sets of treatment data to evaluate the effect of imputation on differential protein abundance analysis. The MDA-MB-468 dataset was filtered to remove lowly expressed protein (protein was removed if total intensity was <2^15^ (32,768). Samples were grouped according to the different treatments (high glucose—HG or glucose deprivation—GD). Protein expression values for simulated replicates were created separately for the HG group and GD groups using the mean and standard deviation of each protein within a group, excluding observations of NA. This simulated dataset was then utilized for testing MAR (*k*NN, MLE or SVD), MNAR (MinDet, MinProb or QRILC) and hybrid (*k*NN + QRILC) imputation methods. Additionally, the final simulated dataset and resulting DEP analyses were used as the ground truth for the comparison of imputation methods. We then proceeded to test if missing values would influence the downstream statistical calculations and FDR by performing data amputation on the simulated data, effectively producing four new datasets with four separate types and amounts of missingness.

Amputed data were created from the simulated data using the ampute function from the mice package in R. Briefly, the missing value pattern was generated for each mechanism (MAR or MNAR) and applied to the simulated data at the appropriate proportion (0.2, 0.5 or 0.8) separately. Next, a random allocation vector consisting of 1′s and 2′s was created to select the missing value pattern (1 = MAR, 2 = MNAR) for the final data matrix. This was performed for 4 different proportions of missingness by altering the proportion of MAR: MNAR in the following combinations: 0.2: 0.2, 0.2: 0.8, 0.5: 0.5, 0.8: 0.2. Source code for these data is included as R scripts in Supplementary Materials (Supplemental File 1).

### 4.4. Data Processing, Imputation and Differential Expression Analysis of Simulated Datasets

Prior to imputation and differential expression/enrichment analysis, the MAR functions (*k*NN, MLE and SVD) in the imputeLCMD R package [6,37] were altered slightly to allow the random seed generator to freely sample imputation values within consecutive iterations of that method. Additionally, the first element of the model selector that flags the data as a ‘1’ for MAR or ‘0’ for MNAR was replaced with a vector of ‘1 s’ so that all MAR values would be imputed. Data were processed by Selection-Filter-Imputation (SFI Method). During selection, data columns were first grouped by sample or treatment type and then chosen for the appropriate pair-wise comparisons. Lowly expressed/enriched proteins were removed from the data set if the minimum number of observations was <3 and total intensity was <2^15^. Missing values were imputed as all MAR (*k*NN, MLE or SVD), all MNAR (MinDet, MinProb or QRILC) or MAR/MNAR (*k*NN + QRILC) for 25 consecutive iterations. For the MAR methods, the model selector was converted to disallow MNAR imputation values from occurring. The typical model selector was utilized during the MAR/MNAR strategy. However, it is important to note imputation was performed separately within each treatment or group of data. Therefore, we introduced this as a hybrid method. Quantile normalization was performed, and limma was used to determine DEPs. Specifically, significance was determined with a modified Student’s *t*-test using the decideTests function in limma that uses a Bayesian model to moderate standard error across proteins [40]. The calculated *p*-value is adjusted for multiple testing by Benjamini-Hochberg correction. To control the FDR, the resulting adjusted *p*-values are converted to *q*-values with the *q*-value function. The protein list from the differential expression analysis was then ordered by *q*-value in ascending order (rank-ordered by *q*-value). Rank-ordering by -*p*-value was used by many labs, including our own, to aid in downstream informatic analysis and validation of potential targets [3,41,42,43]. However, to assess how imputation affects the FDR, we chose to determine the *q*-value and use it as a standard for thresholding [44,45,46,47]. A *q*-value cut-off of less than 0.05 was designated as a significant difference. 

### 4.5. Data Processing, Imputation, Differential Expression/Enrichment Analysis and Top Protein Lists with Proteomic Data

Proteomic data from MDA-MB-468 and NT2 IPs were processed and analyzed as described in the simulated and amputed methods section above. Following DE analysis, datasets from each method were rank-ordered by *q*-value mean to generate the top 200 protein lists. Spread plots were generated from the same rank-ordered lists: the data were filtered for the top 10 proteins from each method and merged into one common list, retaining all unique identifications and removing duplications.

### 4.6. Relative Ranking of Analyses

To compare each method, we chose to assess the relative *q*-value ranking for canonical PRC2 complex members (AEBP2, EED, EZH2, JARID2, PCL (MTF2 in this case), RbAp46 and SUZ12) as all of these proteins were identified in both IP analyses. The protein list from each DEP analysis was first rank-ordered by *q*-value mean, with the lowest *q*-value being the highest rank. For each component of the PRC2 complex, the *q*-value ranking (overall position) was used to determine the rank of each protein across all the methods. For a given protein (row), the method with the lowest *q*-value was rated as 1, the next lowest a 2, and so on for 3 through 7. We reported the rank for the PRC2 complex of proteins across all methods by adding the ratings down each column. The final rating was determined by summing the ranking totals of both IPs (adding the 7 method columns for both IPs together). All source code for this investigation was completed with various packages using R (version 3.6.2) [48] and is available in Supplemental Materials (Supplemental File S1).

## 5. Conclusions

In summary, we explored MAR (*k*NN, SVD, MLE), MNAR (MinDet, MinProb, QRILC) and SFI-hybrid (*k*NN + QRILC) missing value imputation strategies used in intensity-based proteomics workflows. We evaluated the performance of these methods with simulated and real data while considering the extreme ends of missing values (small number of missing values with similar protein expression profiles and a large number of missing values in presence/absence proteomics) encountered in this type of bottom-up proteomics data analysis. From this investigation, we can conclude:

- Single MAR or MNAR strategies are acceptable approaches in proteomics only when the nature of missingness is known to the researcher;
- When an entire protein observation is missing from a treatment or group (three missing values in this case), a single MAR or MNAR imputation strategy is not recommended as the downstream statistics demonstrate the majority of significant identifications contain no missing values. This observation suggests that either the methods have a bias to choose complete cases, or the algorithms are imputing values too close to the observed to be considered significant;
- The statistics with single MAR or MNAR strategies (not the SFI-hybrid) are negatively impacted by increasing number and type of missingness, characterized by large standard deviations, logFC sign fluctuations and an overall trend toward non-significance as seen by the loss in the number of significant proteins from the ground truth and known protein complex interactors;
- To avoid unnecessarily excluding data as in a complete case analysis, a combinatorial MAR/MNAR approach, such as SFI-hybrid, that imputes missing values separately for each treatment group most accurately and reproducibly models bottom-up proteomics data regardless of the missing value type (with the exception of high MNAR as explained in the discussion section).

## Figures and Tables

**Table 1:** Metrics for simulated and one amputed dataset by imputation method. MDA-MB-468 data were simulated as described in the methods section, and shown is the number of differentially expressed (DE) proteins (Sig IDs, *q*-value < 0.05) and the percentage of missing values (% MV) by treatment group (Note: the total number of potential observations in the dataset is 3807 proteins × 6 or 22842). The amputed metrics are from the High MAR instance, where the % Sig IDs is the percentage of the original Sig IDs captured after imputation and DE analysis. GD = glucose deprivation treatment group, HG = high glucose.

Simulated Dataset					Amputed Data						
					High MAR (0.8 MAR: 0.2 MNAR)						
Method	% MV GD	% MV HG	% MV Total	Sig IDs	% MV GD	% MV HG	% MV Total	Sig IDs	% Sig IDs	Sig Lost	Sig Gain
*k*NN	12.2	6.2	9.2	2570	MAR 6.5 MNAR 1.5 TOTAL 8.0	MAR 7.0 MNAR 1.6 TOTAL 8.6	MAR 6.8 MNAR 1.5 TOTAL 8.3	1223	47.6	1368	21
MLE				1794				1256	70.0	583	45
SVD				2494				1601	64.2	916	23
MinDet				2647				1378	52.1	1276	7
MinProb				2527				1273	50.4	1267	13
QRILC				2509				1166	46.5	1347	4
SFI-Hybrid				2537				2386	94.1	254	103

## Data Availability

Proteomics datasets utilized in this study can be downloaded from PRIDE (accessed 3 on June 2019, https://www.ebi.ac.uk/pride/) with the following accession numbers: PXD013966 and PXD004462. Additionally, source code and R packages for this analysis are available through a version-controlled environment in a Binder notebook that can be launched through Git at (https://github.com/mlgred1205/Hybrid_Imputation_for_Missing_Values).

## Supplementary Materials

The following are available online at https://www.mdpi.com/article/10.3390/ijms22179650/s1, Figure S1: MDA-MB-468 whole cell proteomics logFC protein expression, Figure S2: MDA-MB-468 whole cell proteomics −log10 *q*-value mean, Figure S3: EZH2 IP logFC protein expression, Figure S4: EZH2 IP −log10 *q*-value mean, Figure S5: EZH2 IP missingness distribution of top 100 proteins, Figure S6: EZH2 IP spread plots of −log10 *q*-value mean vs. logFC, Figure S7: SUZ12 IP spread plots of −log10 *q*-value mean vs. logFC, Figure S8: SUZ12 IP logFC protein expression, Figure S9: SUZ12 IP −log10 *q*-value mean, Figure S10: SUZ12 IP missingness distribution of top 100 proteins, Table S1: Simulated and amputed data metrics including number and distribution of missing values and number of differentially expressed significant proteins by imputation and amputation methods, Table S2: Ranking of PRC2 proteins identified in EZH2 and SUZ12 IPs, Table S3: MDA-MB-468 whole cell proteomics merged top 10 protein list across the 6 imputation methods, Table S4: EZH2 IP merged top 10 protein list across the 6 imputation methods, and Table S5: SUZ12 IP merged top 10 protein list across the 6 imputation methods.

## Abbreviations

DDA	data-dependent acquisition
MAR	missing at random
MNAR	missing not at random
LOD	limit of detection
MCAR	missing completely at random
*k*NN	*k* nearest neighbors
MLE	maximum likelihood estimation
SVD	singular value decomposition
IP	immunoprecipitation
IgG	immunoglobulin
MinDet	deterministic minimum
MinProb	probabilistic minimum
QRILC	quantile regression imputation of left-censored data
DEP	differentially expressed/enriched proteins
MI	multiple imputation
MI-MFA	multiple imputation in multi-factor analysis
PRIDE	PRoteomics IDEntifications
NT2	NTERA2 cells
PRC2	polycomb repressive complex 2
FDR	false discovery rate
LFQ	label-free quantitation
GD	glucose deprivation
HG	high glucose
SFI	select filter imputation
IP-MS/MS	immunoprecipitation tandem mass spectrometry
