# Supplementary material for: Multiple Imputation Approaches Applied to the Missing Value Problem in Bottom-Up Proteomics
Source: Int J Mol Sci. 2021 Sep 6;22(17):9650. doi: 10.3390/ijms22179650 (PMC8431783; doi:10.3390/ijms22179650)
Supplement: Supplementary file 1 [file ijms-22-09650-s001.zip › Supplemental Figures_IJMS.pdf]

TITLE

Multiple Imputation Approaches Applied to the Missing Value Problem in Bottom-up Proteomics

AUTHORS

Miranda L. Gardner[1,2] and Michael A. Freitas*[1,2]

[1]Ohio State Biochemistry Program, The Ohio State University, Columbus Ohio

[2]Cancer Biology and Genetics, Wexner Medical Center, The Ohio State University, Columbus, Ohio

*Corresponding author email: freitas.5@osu.edu

TABLE OF CONTENTS

Figure S1. MDA-MB-468 whole cell proteomics logFC protein expression

Figure S2. MDA-MB-468 whole cell proteomics –log10 *q*-value mean

Figure S3. EZH2 IP logFC protein expression

Figure S4. EZH2 IP –log10 *q*-value mean

Figure S5. EZH2 IP missingness distribution of top 100 proteins

Figure S6. SUZ12 IP spread plots of –log10 *q*-value mean vs logFC

Figure S7. SUZ12 IP logFC protein expression

Figure S8. SUZ12 IP –log10 *q*-value mean

Figure S9. SUZ12 IP missingness distribution of top 100 proteins

Table S2. Ranking of PRC2 proteins identified in EZH2 and SUZ12 IPs

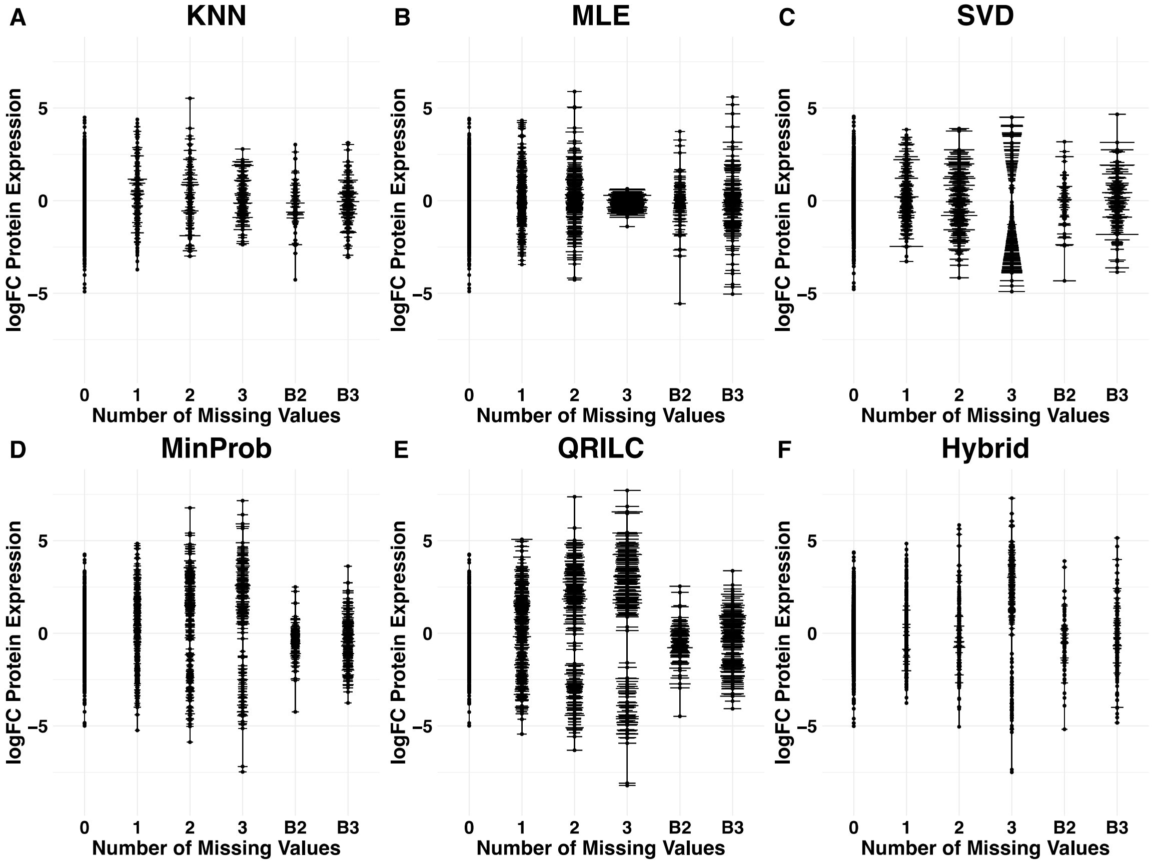

Supplemental Figure S1. Range of logFC protein expression as a function of the number and type of missing values in MDA-MB-468 following 25 consecutive iterations of each imputation method. The number of missing values can be missing in one sample group as 0, 1, 2 or 3 or in any combination of both sample groups as B2 or B3. The horizontal lines represent the standard deviation in the logFC values across the multiple imputations scaled by a factor of 0.5.

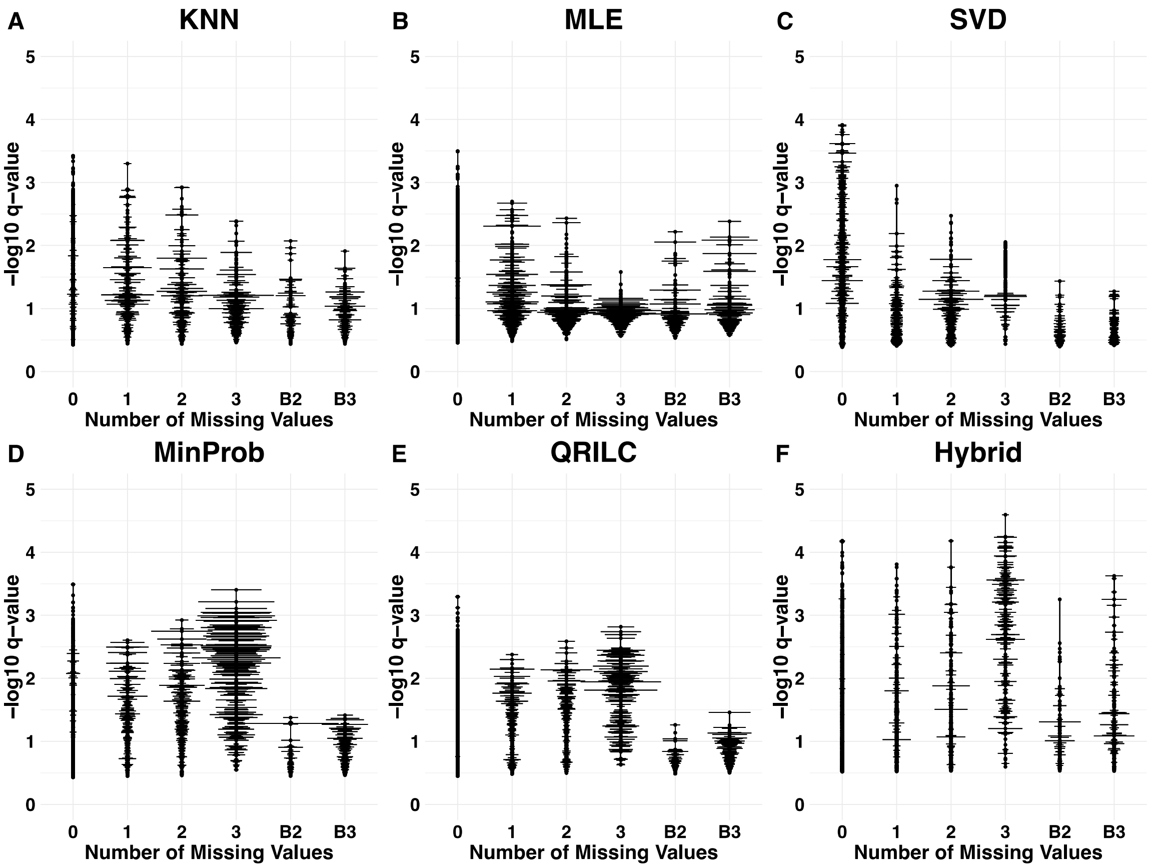

Supplemental Figure S2. Range of -log10 *q*-value mean as a function of the number and type of missing values in MDA-MB-468 following 25 consecutive iterations of each imputation method. The number of missing values can be missing in one sample group as 0, 1, 2 or 3 or in any combination of both sample groups as B2 or B3. The horizontal lines represent the range of *q*-value means across the multiple imputations scaled by a factor of 0.5.

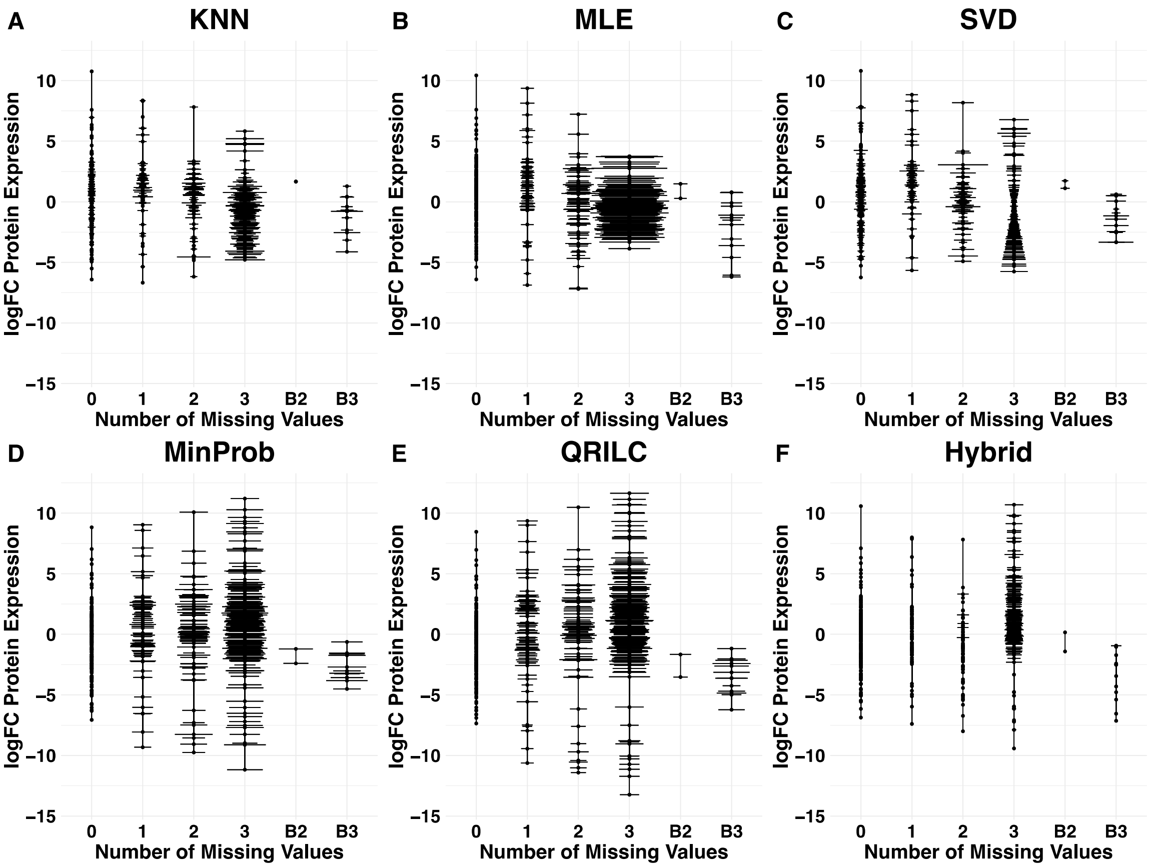

Supplemental Figure S3. Range of logFC protein expression as a function of the number and type of missing values in EZH2 IP compared to IgG control. Data was processed as described in Supplemental Figure S1.

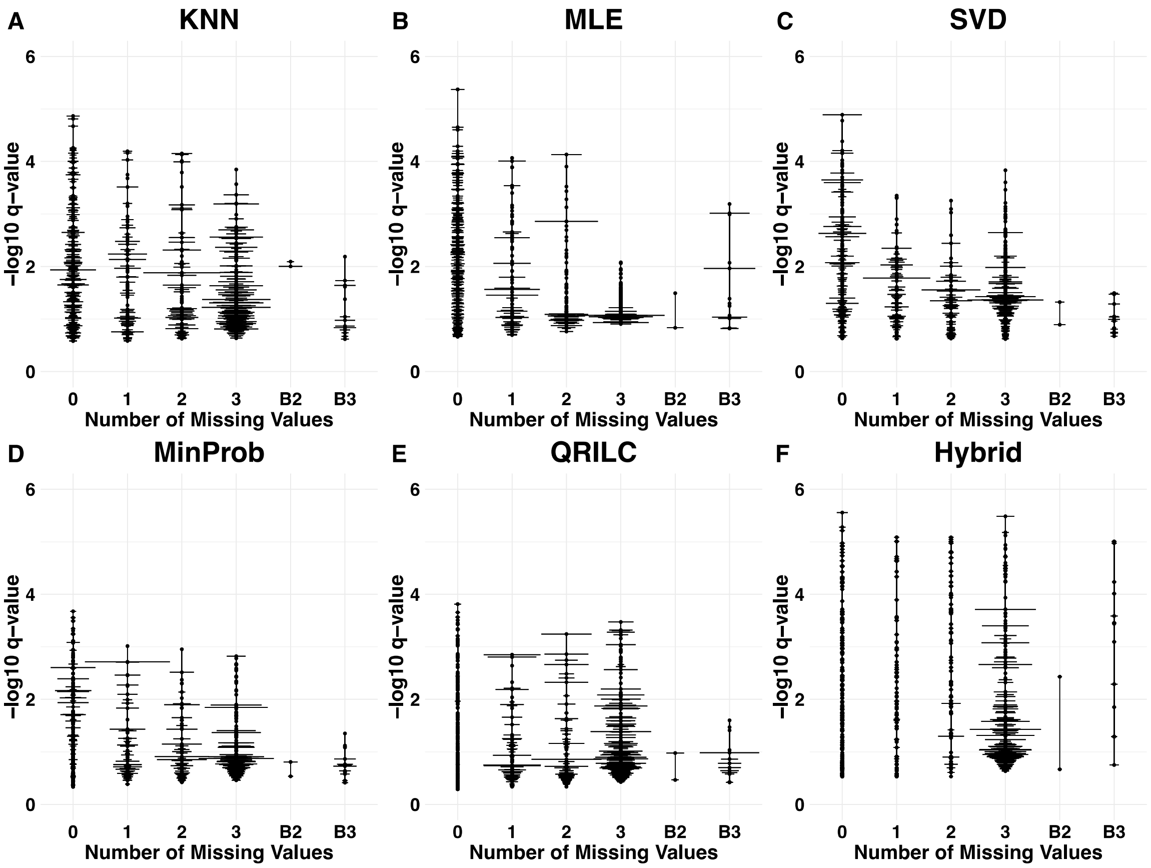

Supplemental Figure S4. Range of -log10 *q*-value mean as a function of the number and type of missing values in EZH2 IP compared to IgG control. Data was processed as described in Supplemental Figure S2.

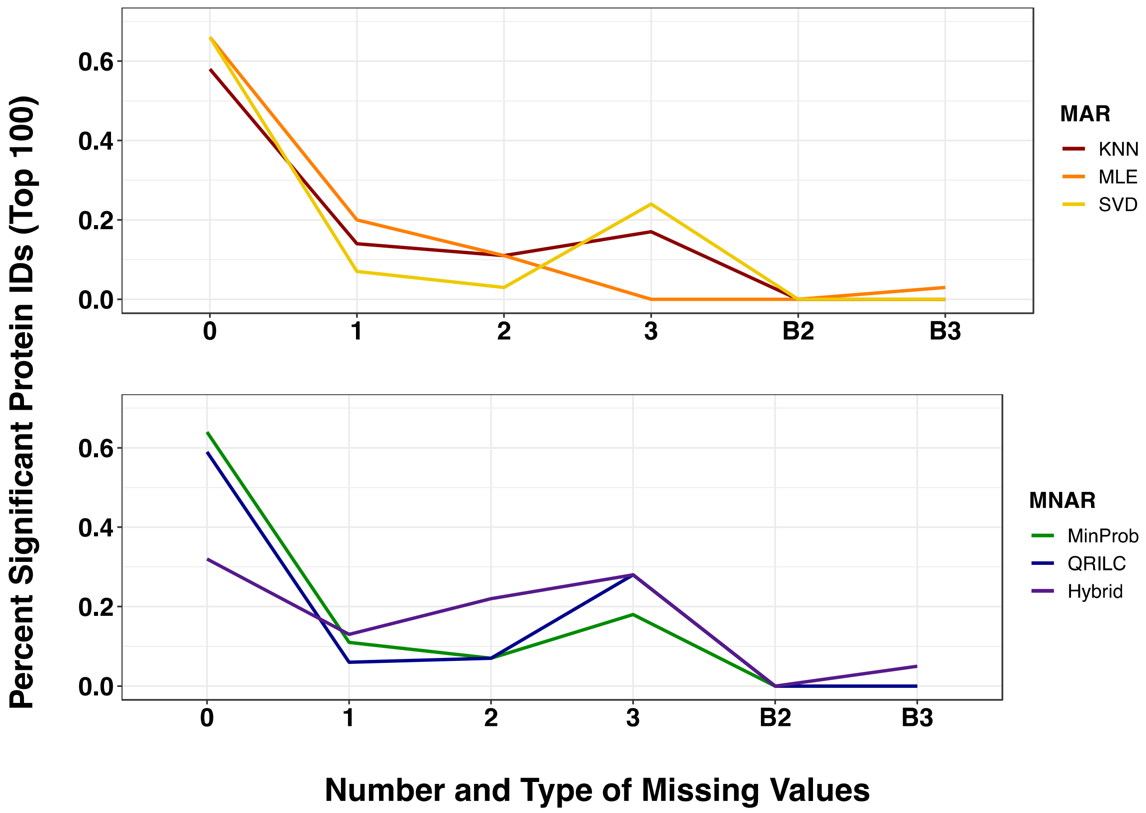

Supplemental Figure S5. Distribution of missingness across all imputation methods with the top 100 significant proteins identified in EZH2 IPs (n = 3). Data was processed as described in Figure 3.

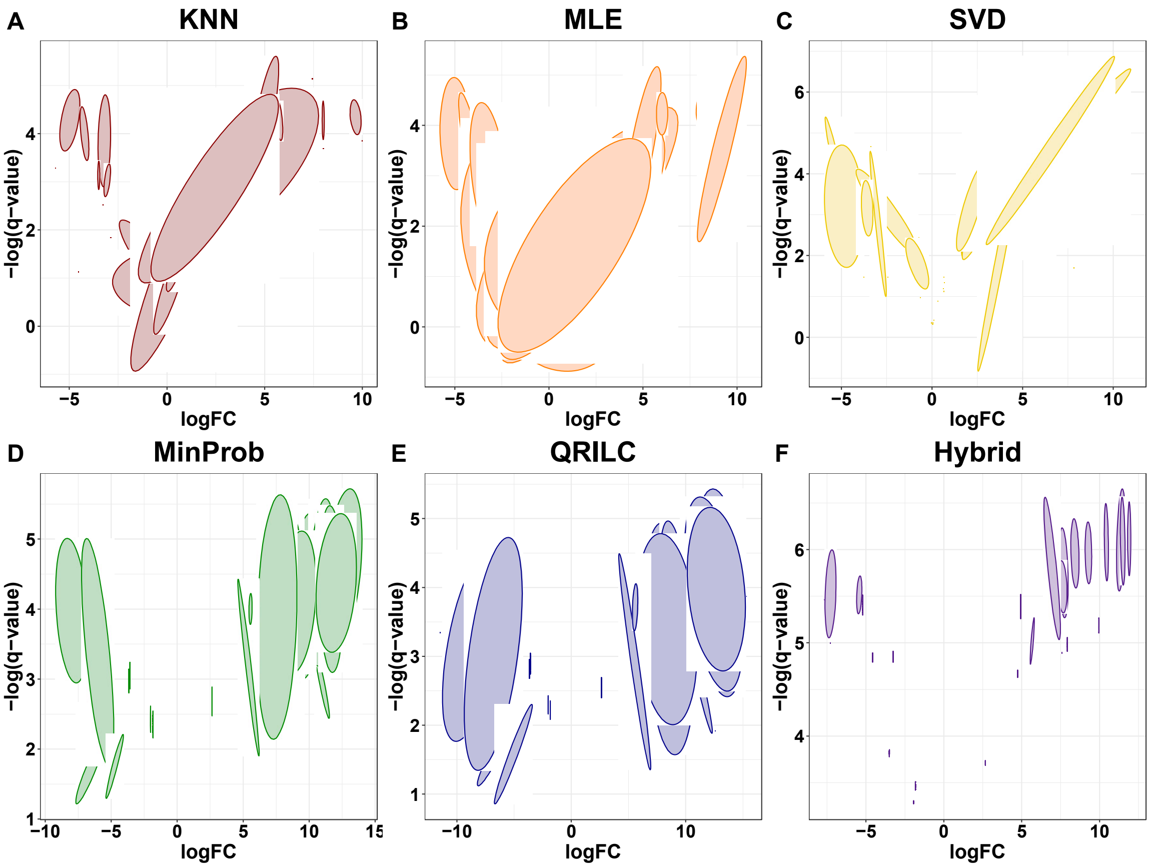

Supplemental Figure S6. Spread plots of -log *q*-value vs logFC for merged top proteins (**Supplemental Table S4**) across all imputation methods for SUZ12 IP data. Data was processed as described in Figure 4.

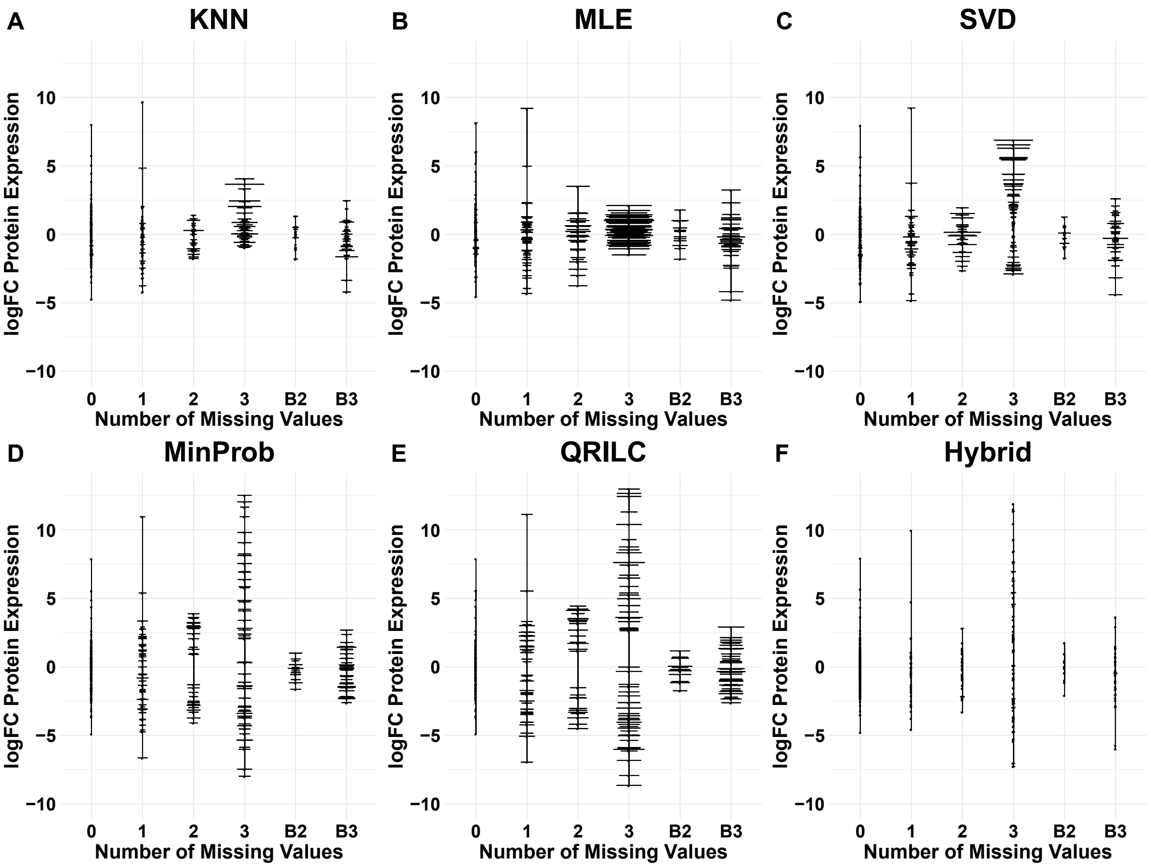

Supplemental Figure S7. Range of logFC protein expression as a function of the number and type of missing values in SUZ12 IP compared to IgG control. Data was processed as described in Supplemental Figure S1.

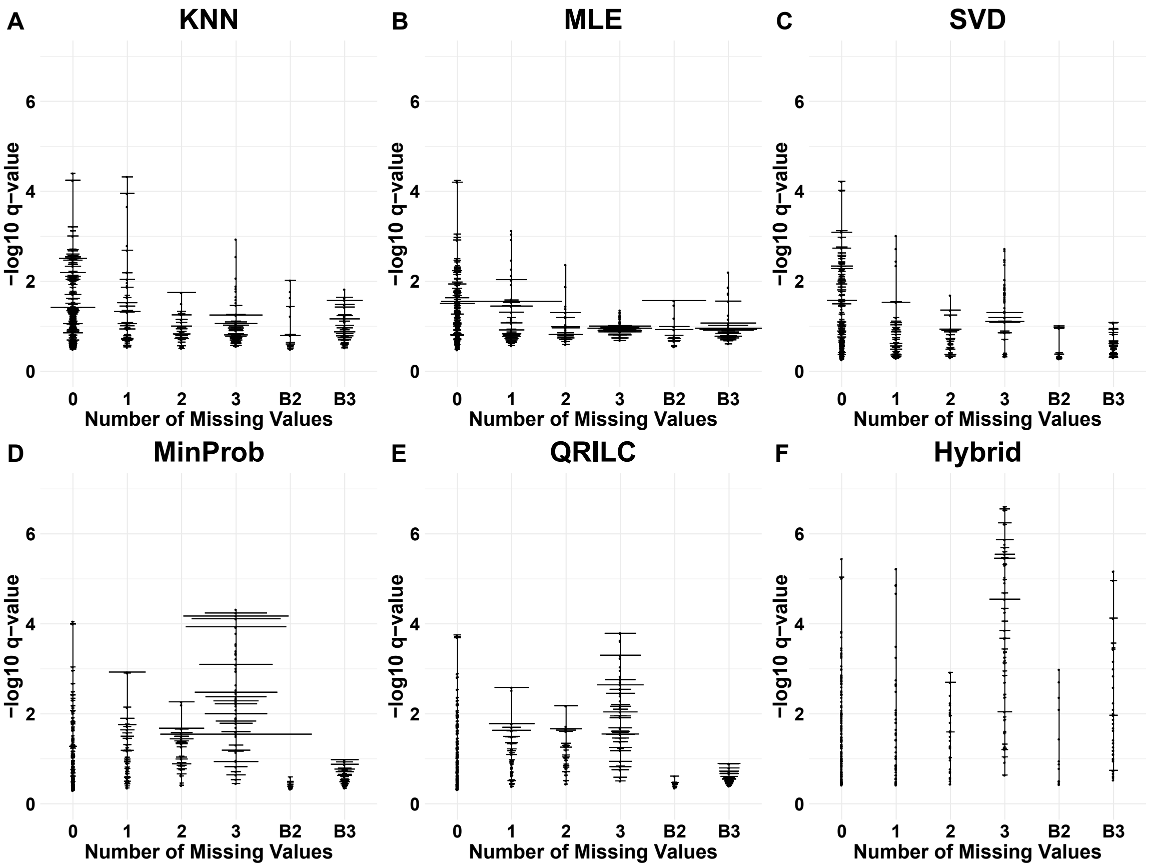

Supplemental Figure S8. Range of -log10 *q*-value mean as a function of the number and type of missing values in SUZ12 IP compared to IgG control. Data was processed as described in Supplemental Figure S2.

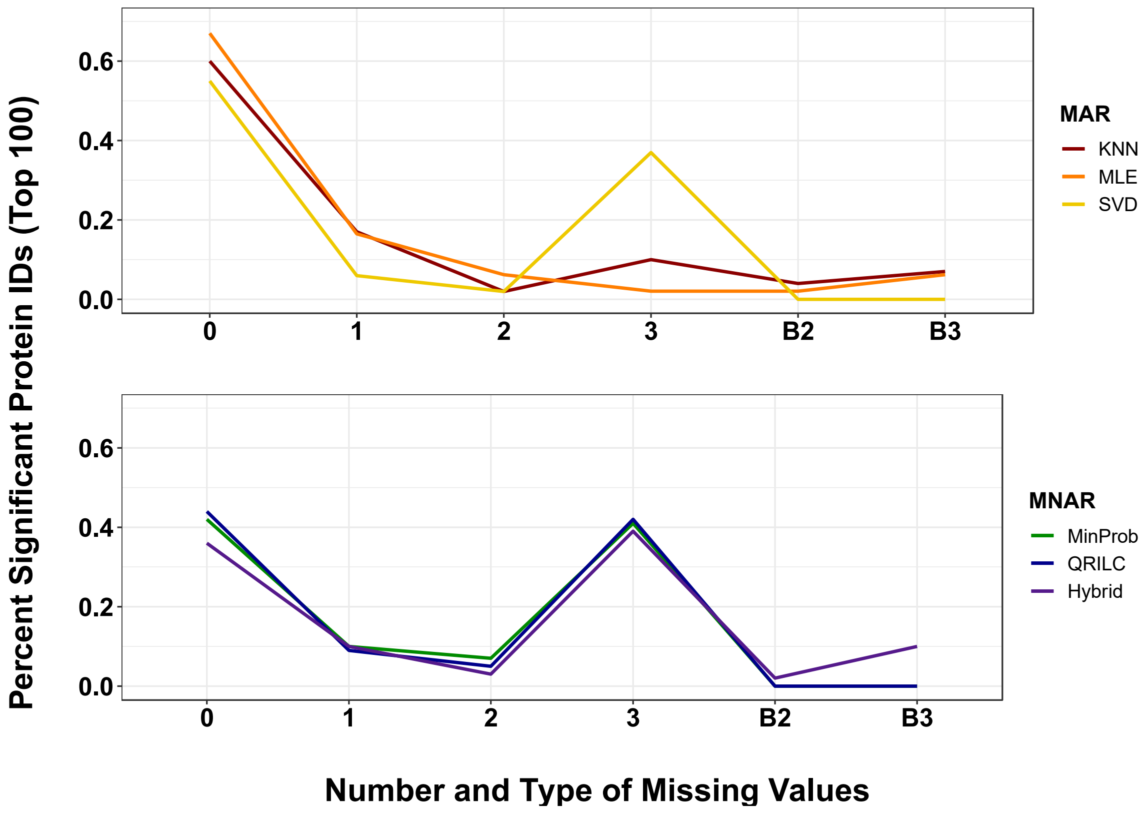

Supplemental Figure S9. Distribution of missingness across all imputation methods with the top 100 significant proteins identified in SUZ12 IPs (n = 3). Data was processed as described in Figure 3.

A

PRC2 Protein	EZH2 KNN	EZH2 MLE	EZH2 SVD	EZH2 MinDet	EZH2 MinProb	EZH2 QRILC	EZH2 Hybrid
AEBP2	62	158	42	6	35	56	46
EED	20	4	33	38	4	4	18
EZH2	24	176	12	1	26	30	6
JARID2	125	216	18	3	34	44	17
PCL	127	157	24	7	42	29	21
RbAp46	455*	328	150	13	76	42	41
SUZ12	8	18	34	120	31	78	10

B

PRC2 Protein	SUZ12 KNN	SUZ12 MLE	SUZ12 SVD	SUZ12 MinDet	SUZ12 MinProb	SUZ12 QRILC	SUZ12 Hybrid
AEBP2	163*	128*	56	2	3	1	4
EED	3	1	3	22	5	3	22
EZH2	10	125*	27	1	1	6	1
JARID2	43	162*	61	3	4	7	3
PCL	58	135*	63	4	2	2	2
RbAp46	209*	173*	20	12	12	16	9
SUZ12	2	6	6	41	26	22	18

Supplemental Table S1. Ranking of PRC2 proteins identified in the IPs. EZH2 (**A**) and SUZ12 (**B**) IPs were rank-ordered by *q*-value mean for each imputation method following consecutive iterations and the final position out of all protein identifications within each method was recorded.

* designates non-significance at a *q*-value threshold cutoff < 0.05.
